# Clinical TNM Lung Cancer Staging: A Diagnostic Algorithm with a Pictorial Review

**DOI:** 10.3390/diagnostics15070908

**Published:** 2025-04-01

**Authors:** Ivana Kuhtić, Tinamarel Mandić Paulić, Lucija Kovačević, Sonja Badovinac, Marko Jakopović, Margareta Dobrenić, Maja Hrabak-Paar

**Affiliations:** 1 Department of Diagnostic and Interventional Radiology, University Hospital Centre Zagreb, 10000 Zagreb, Croatia; 2 Department of Pulmonology, University Hospital Centre Zagreb, 10000 Zagreb, Croatia; 3 School of Medicine, University of Zagreb, 10000 Zagreb, Croatia; 4 Department of Nuclear Medicine and Radiation Protection, University Hospital Centre Zagreb, 10000 Zagreb, Croatia

**Keywords:** lung cancer, TNM staging, computed tomography (CT), International Association for the Study of Lung Cancer (IASLC)

## Abstract

Lung cancer is a prevalent malignant disease with the highest mortality rate among oncological conditions. The assessment of its clinical TNM staging primarily relies on contrast-enhanced computed tomography (CT) of the thorax and proximal abdomen, sometimes with the addition of positron emission tomography/CT scans, mainly for better evaluation of mediastinal lymph node involvement and detection of distant metastases. The purpose of TNM staging is to establish a universal nomenclature for the anatomical extent of lung cancer, facilitating interdisciplinary communication for treatment decisions and research advancements. Recent studies utilizing a large international database and multidisciplinary insights indicate a need to update the TNM classification to enhance the anatomical categorization of lung cancer, ultimately optimizing treatment strategies. The eighth edition of the TNM classification, issued by the International Association for the Study of Lung Cancer (IASLC), transitioned to the ninth edition on 1 January 2025. Key changes include a more detailed classification of the N and M descriptor categories, whereas the T descriptor remains unchanged. Notably, the N2 category will be split into N2a and N2b based on the single-station or multi-station involvement of ipsilateral mediastinal and/or subcarinal lymph nodes, respectively. The M1c category will differentiate between single (M1c1) and multiple (M1c2) organ system involvement for extrathoracic metastases. This review article emphasizes the role of radiologists in implementing the updated TNM classification through CT imaging for correct clinical lung cancer staging and optimal patient management.

## 1. Introduction

Lung cancer is the most commonly occurring cancer, with 2.5 million new cases in 2022 worldwide [1]. It is the leading cause of cancer-related death globally, with an estimated 1.8 million deaths in 2022 (18.7% of all cancers globally) [1]. The estimated number of deaths from lung cancer in 2024 was 125,070 in the United States and 237,434 in the European Union [2,3]. Some of the known causative agents of lung carcinoma include active and passive tobacco smoking, genetic susceptibility, poor diet, chronic inflammation, air pollution, ionizing radiation, and exposure to asbestos, radon, or heavy metals [4,5,6]. In addition, pulmonary fibrosis, HIV infection, and alcoholism may also be responsible for increased risk of lung cancer [7,8,9].

Lung cancer is fatal in most cases, as it is usually diagnosed at a late stage when the treatment is rarely curative. Currently, up to 85% of lung cancers are diagnosed in advanced stages [10]. When the symptoms are present, the disease is usually already in an advanced stage, and treatment options are limited. Therefore, the most useful way to reduce mortality from lung cancer, apart from reducing the risks, is its early detection and treatment. Chest low-dose computed tomography (LDCT) is increasingly being used for the early detection of lung cancer in asymptomatic high-risk individuals. According to the American Cancer Society, lung cancer screening is recommended for current or ex-smokers aged 50 to 80 with a ≥20 pack-year smoking history [11]. Different European countries use distinct inclusion and exclusion criteria according to specific regional and national requirements [12]. Due to the implementation of lung cancer screening programs, more lung carcinomas are being detected at all stages. In the United States, distant-stage disease decreased by 4.6% annually and localized disease increased by 3.6% annually between the years 2013 and 2019 [13]. The introduction of the national lung cancer screening program in South Korea decreased overall one-year mortality (−3.21% points, 95% confidence interval (CI) −4.84 to −1.58) and lung cancer-related mortality (−2.69% points, 95% CI −4.24 to −1.13) [14]. As the screening programs go on, cancers are increasingly being found at earlier stages, leading to a stage shift with improved cure rate and survival.

The optimal treatment for each patient is based on clinical staging and tissue sampling and analysis. The tissue sampling is usually performed either by flexible videobronchoscopy with or without endobronchial ultrasound (EBUS) of the mediastinal lymph nodes, or by computed tomography (CT)-guided percutaneous biopsy. For central endobronchial tumors, videobronchoscopy is the preferred sampling method, whereas peripheral lesions are typically biopsied via percutaneous techniques [15,16]. Lesions with a positive CT bronchus sign, i.e., air-filled bronchus nearby the lesion on a CT scan, are more likely to be diagnosed with guided bronchoscopy than lesions without a bronchus sign [17,18]. Tissue sampling provides histological confirmation of lung cancer and analysis of molecular and immune biomarkers [19]. In cases with metastatic disease, a biopsy of the metastatic lesion may also be performed. If a tissue specimen is not available, cytological samples or liquid biopsy may also be obtained [20].

Clinical staging is based on medical history, physical examination, morphological cross-sectional imaging (CT, magnetic resonance imaging (MRI)), metabolic imaging (positron emission tomography/CT (PET/CT)), endoscopy (bronchoscopy, EBUS, endoscopic ultrasound (EUS)), and minimally invasive surgical procedures (mediastinoscopy) [21]. These procedures should be performed sequentially with an increasing degree of invasiveness [22,23]. The use of the recently proposed ninth edition of lung cancer TNM classification started on 1 January 2025.

This review aims to show the diagnostic algorithm for lung cancer staging according to the ninth TNM edition, to provide a comprehensive pictorial essay that could help radiologists to better interpret the imaging findings, and to identify potential pitfalls in imaging interpretation.

## 2. Diagnostic Algorithm

**Figure 1:**
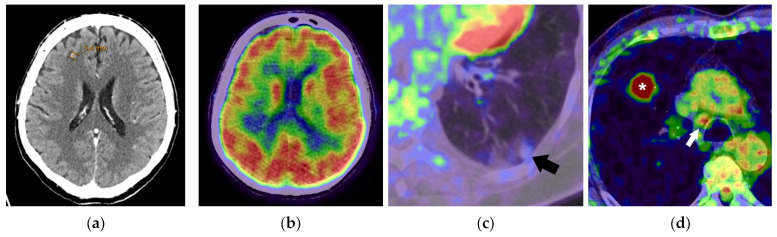
Limitations of FDG PET/CT in lung cancer staging. A small brain metastasis, visible on a contrast-enhanced CT scan in the right frontal lobe (**a**), cannot be detected on an FDG PET/CT scan (**b**) due to the brain’s high physiological glucose uptake. The left lower lobe’s subsolid nodule is not FDG-avid, although it was histopathologically confirmed as adenocarcinoma (**c**). The right lower paratracheal lymph node is FDG-avid (arrow) in a patient with a right-sided lung cancer (asterisk) but was negative at EBUS and as a surgical specimen (**d**).

The stage of proven lung cancer is determined according to the European Society for Medical Oncology (ESMO), American College of Radiology (ACR) Appropriateness Criteria, and American Society of Clinical Oncology (ASCO) by baseline CT examination of the thorax and abdomen and PET/CT examination before initiating therapy [21,24,25]. The ASCO recommends fluorodeoxyglucose (FDG) PET/CT if no distant metastases are detected on a CT scan [25]. This approach has an important benefit in preoperative staging because it may identify hidden distant metastases (commonly found in the liver, adrenal glands, and bones) and helps avoid unnecessary aggressive local treatments in patients with advanced disease [26]. Furthermore, FDG PET/CT is advised for the initial assessment of hilar and mediastinal lymph nodes due to its higher sensitivity of 0.85 and specificity of 0.90, in contrast to CT, which has a sensitivity of 0.61 and specificity of 0.79 [27]. The important limitation of FDG PET/CT is the detection of brain metastases, because the brain has a high physiological glucose uptake (Figure 1). Moreover, FDG PET/CT is not effective for certain cancers with low FDG avidity (e.g., carcinoid tumors, indolent lung adenocarcinomas). Another potential pitfall in PET/CT interpretation is lesions that are FDG-avid but unrelated to lung cancer, like inflammation, infection, and other benign and malignant tumors. The sensitivity of PET is insufficient to properly characterize nodules smaller than 8–10 mm, but the spatial resolution is improved with newer generations of PET/CT scanners [28].

**Figure 2:**
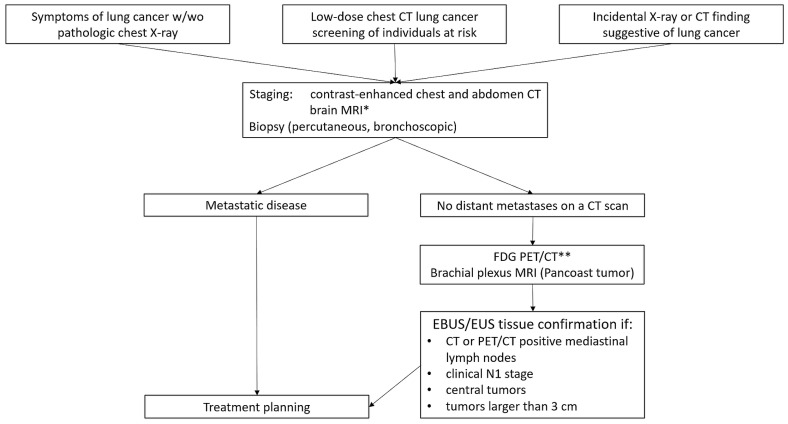
A diagnostic algorithm for a suspected lung cancer. CT—computed tomography, FDG PET/CT—fluorodeoxyglucose positron emission tomography/computed tomography, MRI—magnetic resonance imaging, EBUS—endoscopic bronchial ultrasound, EUS—endoscopic ultrasound. * Brain MRI is not required in all patients with lung cancer (see text). ** FDG PET/CT may be useful in some patients with metastatic disease.

There is some controversy among existing guidelines regarding the evaluation of possible brain metastases by MRI. The ESMO guidelines state that MRI screening for brain metastases might be useful in patients considered for curative therapy [29]. Sometimes, other imaging and invasive diagnostic methods are needed to determine the exact stage, such as chest MRI, bone scintigraphy, or tissue confirmation of mediastinal lymph nodes by EBUS/EUS or mediastinoscopy [21]. The ESMO recommends tissue confirmation of mediastinal lymph nodes in all patients with CT- or PET/CT-positive mediastinal lymph nodes, clinical N1 stage, central tumors, and all tumors larger than 3 cm [21]. A diagnostic algorithm is schematically depicted in Figure 2. The advantages and potential limitations of PET/CT and MRI in lung cancer staging are summarized in Table 1.

Clinical staging determines treatment planning, prognosis, and risk stratification; therefore, accurate imaging interpretation is essential for evaluation of the disease extent. In patients with early or locally advanced lung cancer, curative surgical treatment or radiation therapy is possible, whereas systemic treatment using chemotherapy, immunotherapy, and/or targeted therapy is the main approach in metastatic lung cancer. A detailed knowledge of the staging system, as well as the advantages and limitations of different imaging modalities, is required for optimal patient care. Pathological staging is added to the clinical staging in a subset of patients who undergo surgical resection of the tumor with a proper intraoperative lymph node assessment [30].

The TNM system is used for lung cancer staging. It includes evaluation of the anatomical extension of the primary tumor (T), lymph node metastases (N), and distant metastases to intra- and extrathoracic organs (M). This system is used for the staging of different histological lung cancer subtypes, i.e., non-small-cell lung cancer (NSCLC), small-cell lung cancer (SCLC), and lung neuroendocrine neoplasms. The updates in the recently proposed ninth edition were created using an International Association for the Study of Lung Cancer (IASLC) database including 124,581 patients from 78 different sites across 25 countries diagnosed with lung cancer between the years 2011 and 2019 [16]. The T descriptor remained the same as in the eighth edition of the TNM staging system. In N staging, the N2 descriptor was split into N2a, which includes single-station ipsilateral mediastinal involvement, and N2b, where multiple stations of ipsilateral mediastinal lymph nodes are involved. The M1c descriptor, which indicates multiple extrathoracic metastases, has been divided into M1c1, where one organ system only is involved, and M1c2, where extrathoracic metastases are found in multiple organ systems. Depending on the assessment of the TNM stage and tumor histology with molecular and immune biomarkers, the multidisciplinary team decides on the best modality of treatment [31]. Available treatment modalities include surgery, radiation, chemotherapy, immunotherapy, targeted therapy, and their combination [32]. Modified approaches for the treatment of primary and metastatic lesions with cryoablation, microwave ablation, and stereotactic ablative radiation therapy (SABR) are also available [33,34]. The CT scan used for staging is also considered to be a baseline CT for the response assessment during and after systemic treatment.

## 3. T Descriptors

The T descriptor characterizes the primary tumor by assessing its size; the extent of invasion into adjacent structures; involvement of the main bronchus, carina, and trachea; associated atelectasis or obstructive pneumonitis; and the location of additional tumor nodes to the primary tumor (Table 2). Given the significant changes to the T descriptor introduced in the eighth edition, the ninth edition of the TNM aimed to validate these revisions with the conclusion that the current eighth edition T descriptors should remain in the ninth edition [35]. However, the tumor invasion of adjacent structures is more precisely defined in the ninth as compared to the eighth edition.

Contrast-enhanced chest CT is the preferred imaging modality for evaluating the T descriptor, particularly for determining tumor size, and for identifying the location of the primary tumor and any additional tumor nodules [21,24,25]. However, assessing the extent of invasion into neighboring structures can be particularly difficult in cases of visceral pleural invasion or minimal chest wall or mediastinal invasion [36]. Although not formally included in the clinical guidelines, advanced imaging techniques such as chest MRI can be a valuable adjunct to chest CT in these situations [37,38,39]. If multiple T descriptors apply to a tumor, the highest T category should be selected.

**Table 2:** T descriptors for the TNM-9 clinical classification of lung cancer.

Descriptor	Description [30]	Comment
Tx	Primary tumor cannot be visualized by imaging or bronchoscopy	Tumor proven by the presence of malignant cells in sputum or bronchial washings [30]
T0	No evidence of a primary tumor	
Tis	Carcinoma in situ	
T1	Tumor ≤ 3 cm in the greatest dimension surrounded by lung or visceral pleura, or in a lobar or more peripheral bronchus	No bronchoscopic evidence of invasion more proximal than the lobar bronchus
T1mi	Minimally invasive adenocarcinoma	Solitary adenocarcinoma ≤ 3 cm in the greatest dimension, with a predominantly lepidic pattern and no more than 5 mm solid component in the greatest dimension [30]
T1a	Tumor ≤ 1 cm in the greatest dimension	
T1b	Tumor > 1 cm but ≤2 cm in the greatest dimension	
T1c	Tumor > 2 cm but ≤3 cm in the greatest dimension	
T2		
T2a	Tumor > 3 cm but ≤4 cm in the greatest dimension Invasion of visceral pleura Invasion of an adjacent lobe Involvement of the main bronchus (up to but not including the carina) Tumor associated with atelectasis or obstructive pneumonitis extending to the hilar region, involving either a part of or the entire lung	Visceral pleural invasion should be considered for pleural-attached nodules and pleural-tag nodules [40] PET/CT can better differentiate the obstructing tumor from the tumor-associated atelectasis than CT [26,37]
T2b	Tumor > 4 cm but ≤5 cm in the greatest dimension	
T3	Tumor > 5 cm but ≤7 cm in the greatest dimension Invasion of parietal pleura or chest wall Invasion of the pericardium, phrenic nerve, or azygos vein Invasion of thoracic nerve roots (T1, T2) or stellate ganglion Separate tumor nodule(s) in the same lobe as the primary	The degree of mediastinal penetration by the tumor needed to invade these T3 structures is not counted as T4 [30]
T4	Tumor > 7 cm in the greatest dimension Invasion of the mediastinum, thymus, trachea, carina, recurrent laryngeal nerve, vagus nerve, esophagus, or diaphragm Invasion of the heart, great vessels (aorta, superior or inferior vena cava, intrapericardial pulmonary arteries or veins), supra-aortic arteries, or brachiocephalic veins Invasion of subclavian vessels, vertebral body, lamina, spinal canal, cervical nerve roots, or brachial plexus Separate tumor nodule(s) in a different ipsilateral lobe than that of the primary	Signs of mediastinal invasion: infiltration of the mediastinal fat or structures that indicate the T4 stage, tumor–mediastinum contact length of more than 3 cm, an obtuse angle between the tumor and the mediastinum [37] Signs of vascular invasion: disappearance of the fat layer between the mass and the vessel, the angle between the mass and vessel wall >90°, a stenosis or deformation of the vascular lumen [41,42]

### 3.1. Tumor Size

Since the T descriptor mostly depends on the size of the primary tumor, the precise measurement of tumor size on chest CT is essential (Table 3). For tumor measurement, lung window settings with a sharp filter are recommended on contiguous images with a section thickness of ≤1 mm [43]. The long-axis diameter of the primary tumor should be recorded in the axial, sagittal, and coronal planes, and the longest measurement should be used for the T descriptor’s assessment [43]. Measurements should be reported in centimeters, with the nearest millimeter increments [44]. To ensure consistency, only the nodule core should be measured, and spiculations should be excluded from the measured tumor diameter [45]. Based on the size, solid nodules ≤ 3 cm in the greatest dimension are considered to be T1 tumors (with subclasses T1a ≤ 1 cm, T1b > 1 cm but ≤2 cm, and T1c > 2 cm but ≤3 cm), masses > 3 cm but ≤5 cm are T2 tumors (with subclasses T2a > 3 cm but ≤4 cm and T2b > 4 cm but ≤5 cm), masses > 5 cm but ≤7 cm are T3 tumors, and those > 7 cm in the greatest diameter are T4 tumors. In contrast to T1, where only the tumor size is decisive, the more advanced stages (T2, T3, T4) take into account the tumor size, the extent of invasion into surrounding structures, and the presence and location of lung metastases.

**Figure 3:**
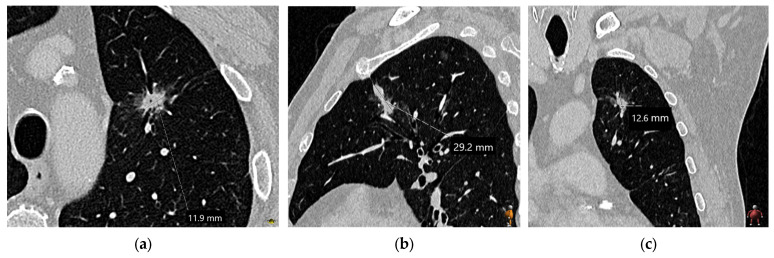
Measurement of solid nodule/mass diameter: The recommended diameter measurement of solid lung cancer on chest CT involves the use of the lung window setting (width 1500, level −600) with the measurement of the longest diameter in the axial (**a**), sagittal (**b**), and coronal planes (**c**). The longest measurement is used for staging; in this case, a sagittal diameter of 2.9 cm indicates the T1c stage. Only the nodule core should be measured, and spiculations should be excluded from the measurement. Measurements for pure ground-glass nodules follow the same principles as measurements for solid nodules.

**Figure 4:**
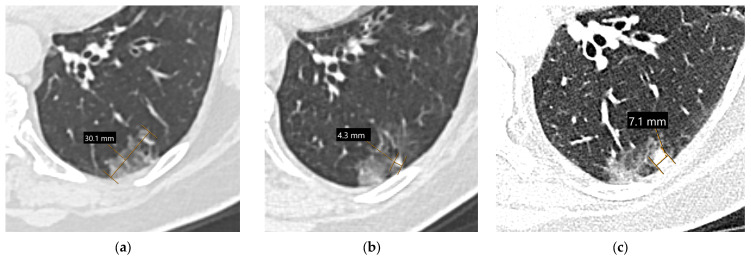
Measurement of subsolid nodule diameter in the lung window setting (width 1500, level −600; the same patient as in Figure 1c): The recommended diameter measurement of the pure ground-glass nodule on chest CT requires measurement of its longest diameter and is classified as Tis if the diameter is between 0.5 cm and 3 cm (**a**). Two years later, the same lesion is partially solid, with the largest dimension of the solid component being 4 mm; therefore the lesion should be categorized as a minimally invasive adenocarcinoma (T1mi, (**b**)). Three years later from the basal scan, the solid component has a diameter of 7 mm (**c**); however, due to visceral pleural invasion, it should be classified as T2a (adenocarcinoma with visceral pleural invasion was confirmed pathologically after lower-left lobectomy).

NSCLC may manifest as a solid, subsolid (including part-solid and pure ground-glass), or cystic nodule or mass on CT imaging, with different measurement strategies. For solid and pure ground-glass nodules, the single largest diameter measured in three standard planes (axial, coronal, and sagittal) should be used (Figure 3). Pure ground-glass nodules that are between 0.5 cm and 3 cm in size are considered to be carcinomas in situ (Tis category, Figure 4a), whereas pure ground-glass nodules larger than 3 cm are classified as T1a [46]. For part-solid nodules, the largest dimension of both the solid and ground-glass components should be measured, and the largest diameter of the solid component should be used for staging (Figure 4b,c), as this has been shown to correlate with invasive adenocarcinomas [44]. Part-solid nodules with a largest diameter of up to 3 cm and a solid component of no more than 5 mm should be categorized as minimally invasive adenocarcinoma (T1mi) [46]. If the solid component exceeds 5 mm in its largest dimension, the subsolid nodule is categorized in the same T category as a solid nodule, based on the largest diameter of the solid component. In cases of part-solid tumors with multiple solid components, only the long axis of the largest solid component should be measured to determine the T descriptor. Although the ground-glass component is often associated with pre-invasive lesions, a significant proportion of pure ground-glass nodules turn out to be invasive adenocarcinomas upon resection [47,48].

**Figure 5:**
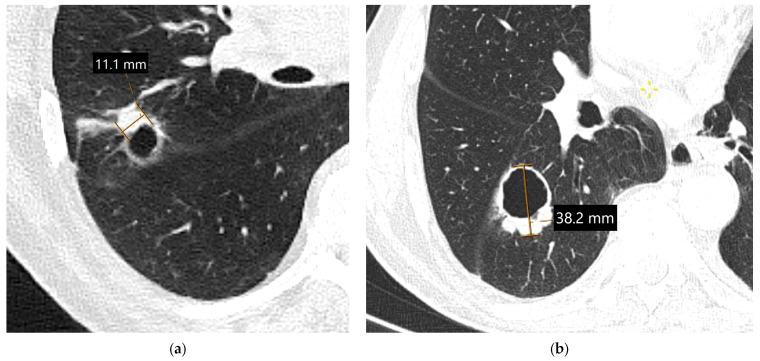
Cystic lung lesion measurement in the lung window setting (width 1500, level −600): In cystic lesions with a peripheral solid nodule, the maximum diameter of the focal solid component should be measured, ignoring the cystic part (**a**). If a circumferential cyst-wall thickening is present, the measurement includes the total lesion, including the cystic airspace (**b**).

There are no clear guidelines for the measurement of cystic lesions, commonly referred to as lung cancer associated with cystic air spaces, and the ninth edition of the TNM classification left unresolved the question of whether to measure the cystic or solid components for staging [43]. If the cystic component of these nodules is included in the overall size of the lesion, the tumor burden may be overestimated, as a substantial part of the lesion consists of air and not solid tumor tissue [49]. Therefore, it may be proposed to measure the maximum diameter of the focal solid component and ignore the cystic part (Figure 5). However, if a circumferential cyst-wall thickening is present, a total lesion size including the cystic airspace might be more suitable [50].

### 3.2. Presence of Atelectasis and/or Post-Obstructive Pneumonitis

**Figure 6:**
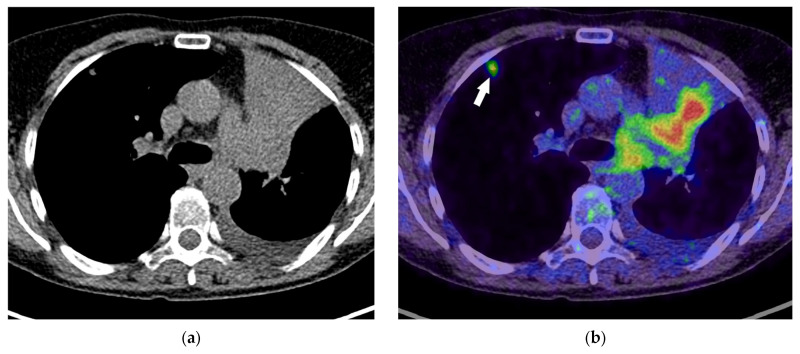
The role of PET/CT in differentiation between obstructive lung cancer and atelectasis: An axial non-contrast-enhanced chest CT image (mediastinal window) showing a left hilar mass accompanied by the atelectasis of the upper-left pulmonary lobe (**a**). A PET/CT image (**b**) confirming an FDG-avid lung cancer in the left hilum and allowing for better differentiation of the tumor from non-FDG-avid upper-left lobe atelectasis (**b**). There is an associated left-sided pleural effusion and an upper-right lobe metastasis (arrow), indicating M1a disease.

When determining lesion size, it is important to consider the presence of adjacent atelectasis and/or post-obstructive pneumonitis. Even for tumors ≤ 3 cm in the greatest dimension, the presence of associated atelectasis or obstructive pneumonitis extending to the hilar region, involving either a part of or the entire lung, should be categorized as T2a. Using chest CT, it might be difficult to find the border between the primary tumor and associated atelectasis. However, accurate localization and measurement of the primary tumor are still crucial for CT-guided biopsy, determination of the radiation field for radiotherapy, and assessment of therapeutic outcomes. In these cases, some degree of estimation is possible with contrast-enhanced CT, but PET/CT or PET/MRI can be valuable additional tools for identifying the obstructing tumor (Figure 6) [26,37].

### 3.3. Involvement of the Main Bronchus, Carina, and Trachea

**Figure 7:**
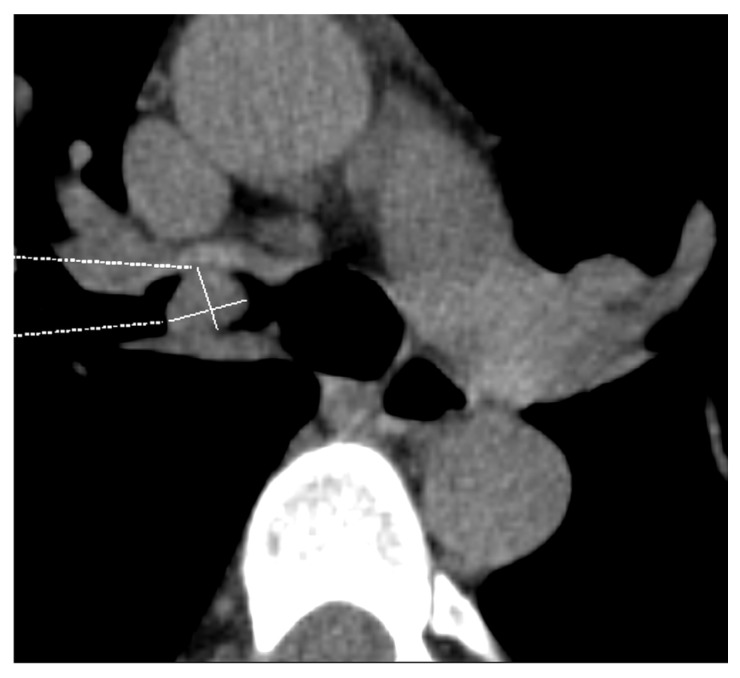
Involvement of the main bronchus: The tumor in the right main bronchus is 1.2 cm in size, which corresponds to stage T1b based on size alone. However, considering the location in the main bronchus (excluding the carina), the tumor should be classified as T2a.

Involvement of the main bronchus, carina, and trachea is best assessed bronchoscopically. Tumors initially classified as T1 are reclassified to the T2a category if they involve the main bronchus, excluding the carina (Figure 7). Any tumor that invades the carina or trachea is classified as T4 disease.

### 3.4. Invasion of Adjacent Structures

#### 3.4.1. T2a Category

Tumors ≤ 3 cm in the greatest dimension are reclassified to the T2a category if they demonstrate visceral pleural invasion or invasion of an adjacent lobe.

**Figure 8:**
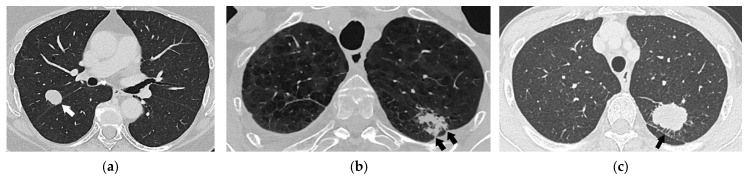
Visceral pleural invasion (T2a), lung window setting (width 1500, level −600): Lung cancer associated with the visceral pleura is categorized into two types: pleural-attached nodules (**a**), which are in direct contact with the pleural surface on imaging (arrow), and pleural-tag nodules (**b**), which are connected to the pleural surface by one or more linear tags (arrows) visible on CT but do not directly touch it. The ‘jellyfish sign’ (**c**) is a hallmark of visceral pleural invasion, characterized by multiple linear septations between the nodule and the pleura (arrow) that resemble jellyfish tentacles. Visceral pleural invasion is confirmed histopathologically in all cases.

Visceral pleural invasion (VPI) is a well-established unfavorable prognostic factor in NSCLC that significantly affects survival, especially in stage IA patients [51]. Consequently, according to the eighth and ninth editions of the TNM staging system, the presence of VPI leads to an upstaging of tumors with a diameter of 30 mm or less to T2a [35]. Lobectomy is superior to sublobar resection in terms of cancer-specific outcomes for NSCLC with VPI [52,53,54]. This highlights the importance of accurate preoperative evaluation of VPI, especially in tumors up to 30 mm in diameter, to guide staging and treatment decisions, as the presence of VPI significantly influences the treatment approach. Medical imaging plays an important role in the VPI assessment; however, its accuracy in detecting VPI is not yet sufficiently reliable. It is rare in small subsolid lung nodules and absent in pure ground-glass nodules; however, it occurs in approximately 25% of subpleural solid nodules [55,56]. NSCLC nodules associated with VPI are generally divided into two categories: pleural-attached nodules and pleural-tag nodules. Pleural-attached nodules directly contact the pleural surface on CT imaging, whereas pleural-tag nodules do not show direct abutment of the pleural surface but have at least one linear tag connecting the nodule to the pleural surface (Figure 8). VPI is observed more frequently in pleural-attached nodules (31%) than in pleural-tag nodules (16%) [40]. For pleural-attached nodules, the most important indicators of VPI on chest CT are the presence of the jellyfish sign (i.e., multiple linear septations between the nodule and pleura, mimicking jellyfish tentacles, Figure 8c), thickening of the pleura, and an increased contact surface area. In pleural-tag nodules, the presence of multiple linear tags to different pleural surfaces is associated with a higher probability of VPI [40].

**Figure 9:**
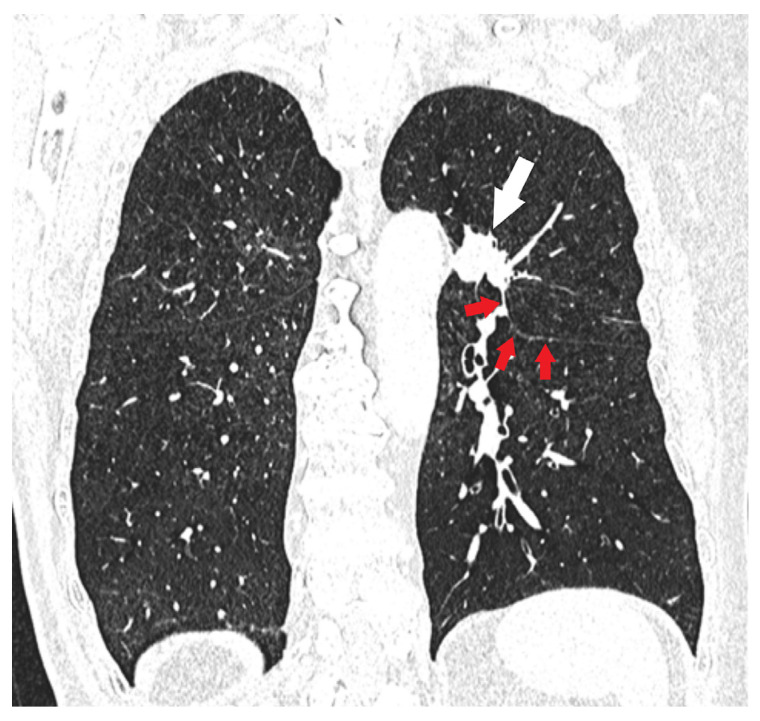
Tumor invading an adjacent pulmonary lobe (T2a): The upper-left lobe lung cancer (white arrow) with an extension through the interlobar fissure (red arrows) into the lower-left lobe should be classified as T2a even if its diameter is 30 mm or less.

According to the ninth TNM edition for lung cancer, tumors invading an adjacent pulmonary lobe are classified as T2a (Figure 9). However, some studies suggest that NSCLC patients with tumors invading the fissure to the adjacent lobe have a similar prognosis as patients classified as having T3 disease, and they propose classifying NSCLC invading an adjacent lobe as T3 disease [57]. Patients with a tumor invading an adjacent lobe have a better prognosis if the transgressed fissure is incomplete as compared to those with a complete fissure at the level of tumor invasion point [58]. Lobectomy with systematic lymph node dissection is insufficient to obtain a complete tumor resection in the case of adjacent lobe invasion, so the surgical approach in these patients includes lobectomy associated with sublobar resection, bilobectomy (right lung), or pneumonectomy. Invasion across a fissure can be suspected on the axial CT images. However, multiplanar CT reformations may help to confirm tumor fissure transgression and the presence of incomplete or accessory fissures, which are important for surgical planning (Figure 9) [59].

#### 3.4.2. T3 and T4 Categories

**Figure 10:**
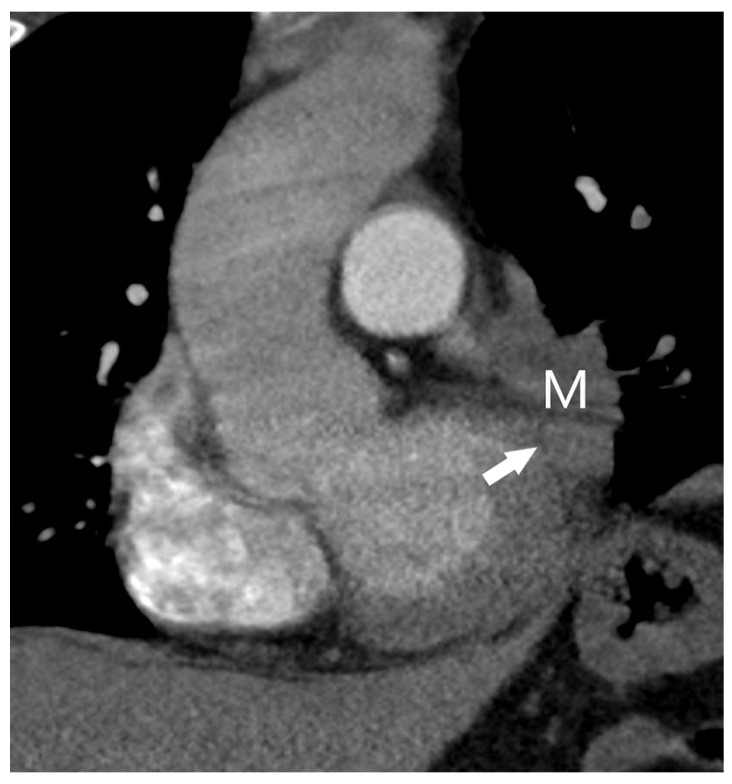
The T3 category: Coronal reformatted CT image (soft-tissue window) showing a mass (M) invading the pericardium (arrow). The left hemidiaphragm is elevated, indicating invasion of the left phrenic nerve.

Tumors smaller than 5 cm should be reclassified into the T3 category if they invade the parietal pleura, chest wall, pericardium, phrenic nerve, azygos vein, thoracic nerve roots (T1, T2), or stellate ganglion. Deeper mediastinal invasion, even for tumors smaller than 7 cm, indicates T4 disease. T4 disease is also present in case of invasion of the thymus, trachea, carina, recurrent laryngeal and vagus nerve, esophagus, heart, mediastinal vessels (aorta, superior or inferior vena cava, intrapericardial pulmonary arteries or veins, supra-aortic arteries, brachiocephalic veins, subclavian vessels), vertebral bodies, laminae, and/or spinal canal. Additionally, the T4 descriptor should be assigned to tumors invading the cervical nerve roots, brachial plexus, or diaphragm. A direct sign of a mediastinal invasion is infiltration of the mediastinal fat or any of the abovementioned structures that indicate the T4 stage. Indirect signs of mediastinal invasion include a tumor–mediastinum contact length of more than 3 cm or an obtuse angle between the tumor and the mediastinum [37]. It is important to note that the pericardium, phrenic nerve, and azygos vein lie within the mediastinum, but the degree of mediastinal penetration by the tumor needed to invade these structures is considered to be T3, not T4 (Figure 10) [30]. Similarly, tumors in contact with the mediastinal pleura without direct or indirect signs of mediastinal invasion should not be automatically staged as T4 [37]. Dynamic MRI sequences have higher sensitivity and specificity for the assessment of mediastinal invasion than a static CT scan [37].

**Figure 11:**
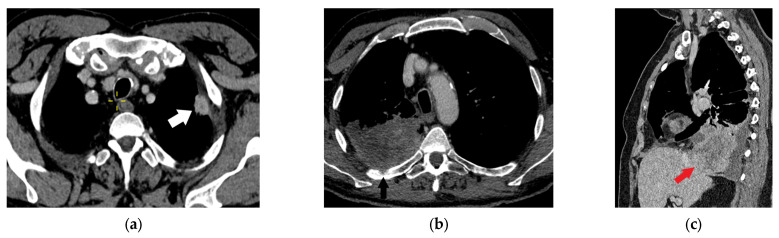
Parietal pleura, chest wall, and diaphragm invasion: Axial contrast-enhanced chest CT image shows lung cancer in the upper-left lobe (white arrow) invading the parietal pleura and extrapleural fat (T3 category, (**a**)). Rib erosion (black arrow) by upper-right lobe lung cancer is a reliable sign of chest wall invasion (T3 category, (**b**)). A sagittal multiplanar reformatted image shows an invasion of the diaphragm (red arrow) by the lower-right lobe lung cancer (T4 category) with associated pleural effusion (**c**).

Assessment of minimal invasion of the parietal pleura, chest wall, or diaphragm can be difficult. However, confirmed involvement of the parietal pleura and chest wall warrants classification as T3, whereas invasion of the diaphragm indicates the T4 category (Figure 11) [37]. Invasion of these structures can usually be assessed well by contrast-enhanced CT, MRI, or PET/MRI. Multiplanar reconstructions are essential for the accurate evaluation of diaphragmatic involvement, as axial reconstructions may underestimate the extent of infiltration. The most reliable signs of chest wall invasion are rib erosions or a broad extension of the tumor into the intercostal space. Impaired respiratory movement or a thickening of the pleura are less reliable signs of the infiltration of neighboring structures, with a limited positive predictive value, because the peritumoral inflammatory reaction may result in the same signs. Since tumors that infiltrate the chest wall and/or diaphragm do not follow normal respiratory movement, dynamic CT and MRI with dynamic CINE sequences can provide a higher accuracy compared to conventional static CT for the evaluation of chest wall infiltration [37]. The assessment of chest wall and diaphragm invasion is difficult with PET/CT due to blooming artifacts [26,28]. If there is a separate pleural nodule from the primary tumor or malignant pleural effusion, the disease is counted as metastatic (M1a).

**Figure 12:**
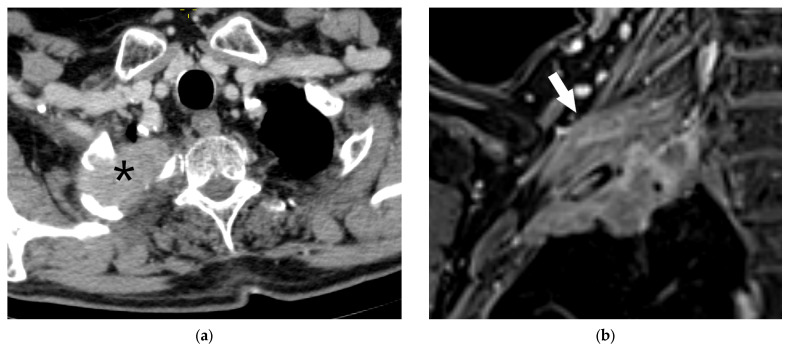
Pancoast tumor: Axial contrast-enhanced chest CT image shows a Pancoast tumor (asterisk) at the upper-right lobe’s apex (**a**), with chest wall invasion and rib erosion. A coronal post-contrast fat-saturated T1-weighted MRI image (**b**) confirms the invasion of the brachial plexus at the C7 to T1 level (arrow, T4 category).

Tumors of the superior sulcus (Pancoast tumors) are classified as at least T3 due to their infiltration of the chest wall (Figure 12). If there is an additional invasion of the brachial plexus above C8, vertebral bodies, the spinal canal, or the subclavian vessels, it should be classified as T4. The cervical and thoracic nerve roots, as well as the stellate ganglion, are located in the posterior compartment of the superior sulcus, between the head of the first rib posteriorly and the vertebral artery anteriorly [60]. The invasion of these structures leads to arm and shoulder pain and Horner syndrome (ipsilateral miosis, eyelid ptosis, and facial anhidrosis). According to the ACR Appropriateness Criteria, MRI assessment of superior sulcus tumors for brachial plexus involvement is a standard of care [24]. Invasion of the cervical and thoracic nerve roots, brachial plexus, or stellate ganglion is better assessed using MRI than by CT, due to its superb soft-tissue contrast resolution [61]. Precise evaluation of the brachial plexus roots is essential if surgical treatment is planned, since the involvement of the plexus above the T1 level is a contraindication for surgical resection [60]. Furthermore, MRI is superior to CT for detecting the involvement of the neural foramina and spinal canal.

**Figure 13:**
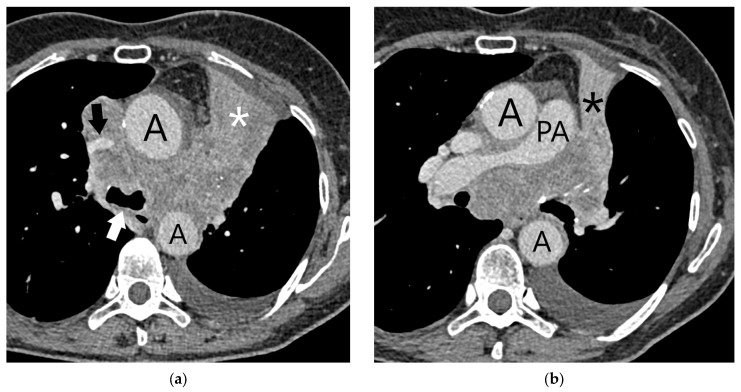
T4 category: Axial contrast-enhanced CT scan of the chest at the level of carina (**a**) and the main pulmonary artery (**b**) shows a T4 category upper-left lobe lung cancer with invasion of the aorta (A), intrapericardial segments of pulmonary arteries (PA), carina (white arrow), and the superior vena cava (black arrow). Additionally, there is upper-left lobe atelectasis (asterisk) and left-sided pleural effusion.

When assessing mediastinal cardiovascular structures, invasion of the pericardium and azygos vein is considered to indicate T3 disease, whereas invasion of deeper mediastinal vessels indicates T4 disease. The azygos vein can be invaded by right-sided lung cancer in its ascending segment along the anterolateral surface of the thoracic vertebrae, or along its arch at the level T5–T6 [62]. In the case of direct pericardial invasion by the primary tumor, there is a high rate of N1 and N2 disease, probably due to rich pericardial lymph drainage [63]. However, if there is a separate pericardial nodule or malignant pericardial effusion, the disease is considered metastatic (M1a). Invasion of great mediastinal vessels is present if there is infiltration of the aorta, superior or inferior vena cava, main pulmonary artery, or intrapericardial segments of the right or left pulmonary artery (Figure 13). The border between the intrapericardial and extrapericardial segments of the right pulmonary artery lies at the mid-half of the superior vena cava, whereas the border of the left pulmonary artery segments is approximately 1 cm distally from the bifurcation of the main pulmonary artery [37]. Involvement of the extrapericardial portion of the pulmonary arteries is not used as a T descriptor; however, it is important to report the invasion of extracardiac pulmonary artery segments by hilar tumors or lymphadenopathy, since it requires technically demanding pulmonary artery resection and reconstruction [64]. The T4 stage is also present if there is an invasion of the heart, supra-aortic arteries, brachiocephalic veins, or subclavian vessels. The criteria for diagnosing cardiovascular invasion using CT include the disappearance of the fat layer between the mass and the vessel/heart, the angle between the mass and vessel wall being >90°, and a stenosis or deformation of the vascular lumen [41,42]. Cardiac involvement can occur either directly by tumor penetration through the mediastinum or indirectly by its extension into the left atrium via the pulmonary vein. Patients with cardiac invasion have a poorer clinical outcome than those without cardiac invasion, so careful preoperative evaluation is mandatory for appropriate patient management [65].

**Figure 14:**
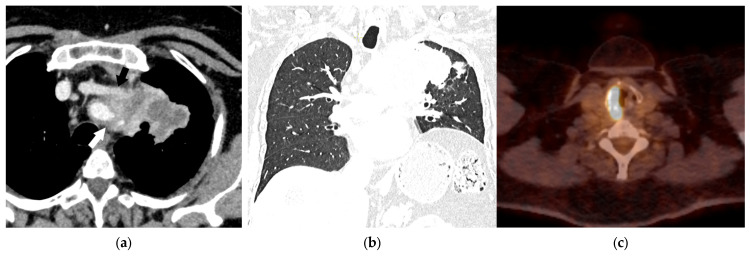
T4 category: Axial contrast-enhanced chest CT image showing lung cancer in the upper-left lobe invading the mediastinum, left brachiocephalic vein (black arrow), and supra-aortic arteries (white arrow, (**a**)). The elevated left hemidiaphragm confirms phrenic nerve invasion or at least the T3 category (**b**). There is increased FDG uptake in the right vocal cord as a result of the left recurrent laryngeal nerve invasion (T4 category), with an increased workload of the right-sided vocal cord muscles (**c**).

Invasion of the phrenic nerve indicates stage T3 and may be recognized by the raised position of the ipsilateral hemidiaphragm due to its paralysis and by invasion along its course from the thoracic inlet to the diaphragm medial to the mediastinal pleura (Figure 10). The phrenic nerve passes anteriorly to the respective pulmonary hilum and inferiorly through a vertical plane between the fibrous pericardium and the mediastinal pleura [66]. If there is an invasion of the aorticopulmonary window by left-sided lung cancer, or of the right brachiocephalic vein or superior vena cava by a right-sided mass, the ipsilateral hemidiaphragm should be evaluated for phrenic nerve involvement [60]. Invasion of deeper mediastinal nerves, including the recurrent laryngeal nerve and the vagus nerve, is indicative of the T4 stage (Figure 14). Invasion of the recurrent laryngeal nerve results in ipsilateral vocal cord paralysis with hoarseness and is more common on the left side due to the longer route of the left recurrent laryngeal nerve, which passes through the aortopulmonary window [67]. In patients with recurrent laryngeal nerve invasion, an asymmetric focal FDG uptake can be noticed in the contralateral vocal cord as a result of the increased workload of the vocal cord muscles (Figure 14c) [68]. Since the vagus nerve is mostly parasympathetic, clinical features of its invasion may be subtle, especially if the invasion occurs below the origin of the recurrent laryngeal nerve. Vagal nerves cross anteriorly to the subclavian artery, and then the right nerve passes through the right paratracheal region, while the left nerve passes laterally to the aortic arch. More caudally, both vagal nerves pass posterior to the lung hilum and enter the abdominal cavity through the esophageal hiatus.

The T4 stage is also present in case of the invasion of the thymus, esophagus, vertebral body, lamina, or spinal canal. In the case of esophageal invasion, patients usually present with dysphagia. On the CT scan, the esophagus may show contralateral displacement or obstruction by the adjacent lung cancer, sometimes with a malignant broncho-esophageal or tracheo-esophageal fistula [69]. A more detailed evaluation of the degree of esophageal wall invasion may be performed using EUS [70]. In comparison with CT, MRI is more accurate for the assessment of invasion of a vertebral body, lamina, or spinal canal [71].

### 3.5. The Location of Additional Tumor Nodules to the Primary Tumor

**Figure 15:**
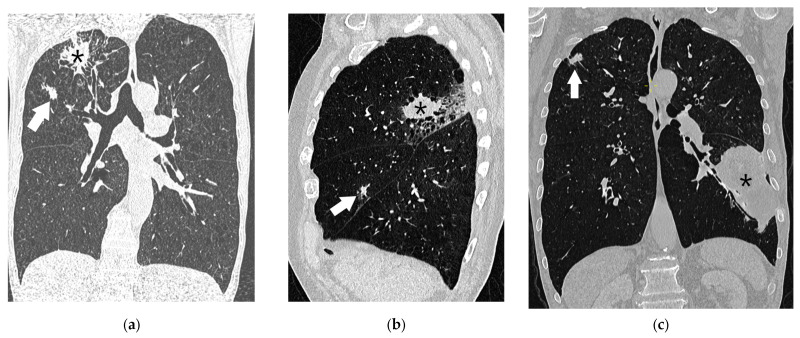
Additional lung nodules in three different patients, lung window setting (width 1500, level −600): A coronal reformatted chest CT image (**a**) showing a primary tumor (asterisk) and a metastatic lesion (arrow) in the upper-right lobe, corresponding to T3. A sagittal reformatted CT image (**b**) showing a primary tumor (asterisk) in the upper-right lobe and a metastatic lesion in the middle lobe (arrow), corresponding to T4. A coronal reformatted CT image (**c**) showing a primary tumor (asterisk) in the lower-left lobe and a metastatic lesion (arrow) in the upper-right lobe, corresponding to M1a.

According to Detterbeck et al., multiple foci can occur in four different patterns in lung cancer patients [72]:

1. If a patient has two separate, histologically different lung cancers, separate TNM staging should be performed for each tumor.
2. In patients with a solid primary lung cancer with one or more separate solid tumor nodule(s) of the same histological type, separate nodules are considered to be intrapulmonary metastases (Figure 15). The presence of separate tumor nodule(s) in the same lobe as the primary tumor indicates T3 disease. If there are separate tumor nodules in a different ipsilateral lobe than that of the primary tumor, stage T4 should be determined. Lung nodules in the contralateral lung indicate distant metastases (M1a disease). Although the classification of multiple lung nodules in lung cancer patients may appear simple, it may lead to overstaging if lesions are not confirmed histologically, since the majority of lung nodules identified in patients with lung cancer are benign [37].

3. Multiple lung cancer nodules with prominent ground-glass or lepidic features should be classified according to the highest T lesion. The lesion size is determined by the largest diameter of the solid component at CT. The number of lesions (#), or simply (m) for multiplicity, should be indicated in parentheses after the T descriptor of the highest lesion. This approach should be applied equally for lesions with ground-glass or lepidic features located in the same lobe or different ipsilateral or contralateral lobes. An N and M category should be applied to all lesions collectively with a single N and M stage.
4. Pneumonic-type lung cancer has a consolidative pattern on CT in the absence of an obstructed bronchus. It should be categorized as T3 if confined to a single lobe, T4 if different ipsilateral lobes are involved, or M1a if contralateral lobes are involved. A T4 designation should be applied both if there is a direct extension into another lobe and when there is a discrete separate area of involvement in an adjacent lobe. Again, a single N and M category should be assigned for all lesions.

## 4. N Descriptors

The N descriptor indicates the intrathoracic extension of the malignant disease into the regional lymph nodes, based on their location using the IASLC lymph node map [73]. This map was created to achieve uniformity by providing precise anatomic definitions for all lymph node stations. The location of affected lymph nodes on multimodal imaging relies on identifying anatomical landmarks, such as the carina, bronchial bifurcations, and vascular structures (Table 4). The subcarinal lymph node is always considered ipsilateral to the primary tumor, whether the primary lung cancer is in the right or left lung [43]. Malignant lymph nodes are identified based on their size on a CT scan, with a short-axis diameter greater than 1 cm considered abnormal. On PET/CT, they are detected by a maximum standardized uptake value (SUV) of 2.5 or greater, or by visual SUV assessment greater than the mediastinal background [27].

**Figure 16:**
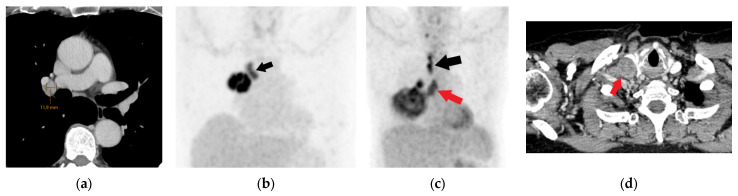
N Descriptors: Axial contrast-enhanced chest CT image showing metastasis in the ipsilateral hilar lymph node (arrow) or N1 disease (**a**). Maximum intensity projection (MIP) FDG PET image showing single-station N2a disease with metastasis in the ipsilateral lower paratracheal station (arrow, (**b**)). MIP FDG PET image in a patient with ipsilateral multiple-station (N2b) metastases in the ipsilateral lower paratracheal (black arrow) and subcarinal (red arrow) stations (**c**). Enlarged supraclavicular lymph nodes (arrow) indicate N3 disease (**d**).

The N0 stage indicates that there is no regional lymph node metastasis (Table 5). The N1 stage includes the involvement of ipsilateral peribronchial, hilar, and intrapulmonary nodes that are located within the affected lung or adjacent to the bronchus, including a direct extension of the primary tumor (Figure 16). The N2 descriptor should be assigned in case of invasion of ipsilateral mediastinal and/or subcarinal nodes, considering the left lateral border of the trachea as the boundary between the right and left paratracheal stations. The ninth edition of the lung cancer TNM classification subdivides the N2 category into N2a and N2b, considering the number of affected regions [74]. The N2a subcategory refers to single-station involvement of ipsilateral mediastinal lymph nodes or subcarinal lymph nodes. The disease is confined to one lymph node group, suggesting a more localized disease that may be eligible for surgery. The N2b subcategory denotes multi-station involvement within the ipsilateral mediastinal and subcarinal lymph nodes, indicating a more advanced disease that has a poorer prognosis and requires systemic therapy. The N3 stage indicates the involvement of contralateral mediastinal nodes, contralateral hilar nodes, or ipsilateral or contralateral scalene or supraclavicular lymph nodes. In patients with SCLC, mediastinal lymphadenopathy is commonly indistinguishable from a hilar primary tumor creating a mediastinal conglomerate mass [75].

The new subdivision of the N2 category is reasonable due to prognostic implications. Patients with pathological single-station N2 disease, compared to those with multiple-station N2 stage, have better overall and recurrence-free survival [77]. Furthermore, the distinction between N2a and N2b has significant implications for clinical decision-making. Patients with N2a disease may benefit from surgical resection as part of a multimodal approach, particularly if other factors suggest operability. By contrast, N2b disease often necessitates systemic therapies, such as chemotherapy or chemoradiation, given the increased likelihood of distant micrometastases. The subdivision also aids in stratifying patients for clinical trials, enhancing precision in staging, and tailoring treatment to individual disease profiles [78].

Changes in N staging reflect the growing importance of imaging technologies, including CT and PET/CT, in identifying and classifying lymph node status [79]. As stated earlier in this manuscript, invasive mediastinal staging using EBUS, EUS, and mediastinoscopy should be performed in all patients with CT- or PET/CT-positive mediastinal lymph nodes, clinical N1 stage, central tumors, and all tumors larger than 3 cm [21]. Furthermore, the aforementioned invasive mediastinal staging techniques might be used to complement imaging modalities in precise differentiation between N2a and N2b disease. Tissue confirmation is usually not required in the case of bulky lymphadenopathy (lymph node diameter larger than 3 cm) with a high probability of lymph node involvement [80,81].

Non-regional intrathoracic lymph node involvement, such as retrocrural, internal mammarian, diaphragmatic, axillary, and intercostal lymphadenopathy, represents distant metastatic disease (M1b or M1c depending on the number of involved lymph nodes) [24,27,76].

## 5. M Descriptors

**Figure 17:**
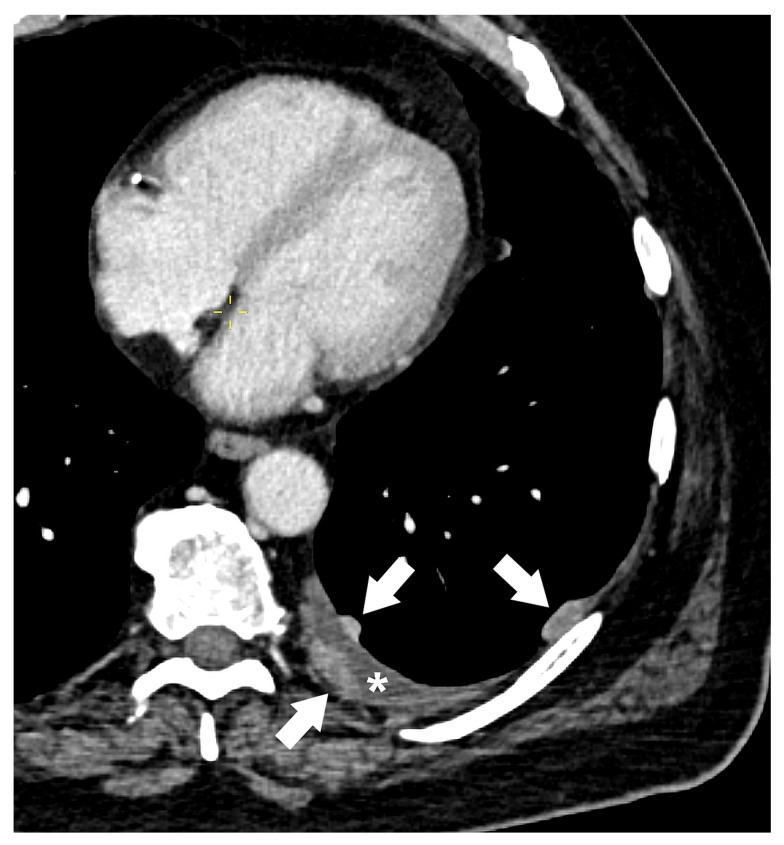
M1a category: Contrast-enhanced CT image showing left-sided pleural enhancing nodules (arrows) with ipsilateral pleural effusion (asterisk), indicating pleural metastases (M1a).

The M descriptor classifies the presence and extent of metastatic disease, indicating the intrathoracic and extrathoracic extension of the malignant disease into other organ systems. According to the eighth edition of the TNM classification, metastatic disease was categorized into four key groups, with further subdivision of M1c into the M1c1 and M1c2 categories in the latest edition (Table 6) [82]. The M0 category represents no evidence of distant metastasis, while the M1a category represents separate tumor nodule(s) in the contralateral lung, pleural or pericardial nodules, or malignant pleural/pericardial effusion. Intrapulmonary metastases typically have similar radiographic appearance, metabolic activity, and growth rates to the primary tumor in comparison with previous imaging, and they may be associated with significant nodal or systemic metastases [37]. If these features are absent, the synchronous primary tumor should be considered. Most pleural and pericardial effusions in patients with lung cancer are caused by the tumor [30]. However, if repeated cytological examinations of pleural/pericardial fluid are negative for cancer cells and the fluid is non-bloody and not an exudate, the effusion is probably not related to the tumor and should be excluded as a staging descriptor. Pleural and pericardial metastases usually manifest on CT as enhancing nodular or lenticular masses, with or without associated pleural/pericardial effusion (Figure 17). Malignant pleural invasion is especially suspicious in cases of nodular pleural thickening, mediastinal pleural thickening, and parietal pleural thickening >1 cm [37].

**Figure 18:**
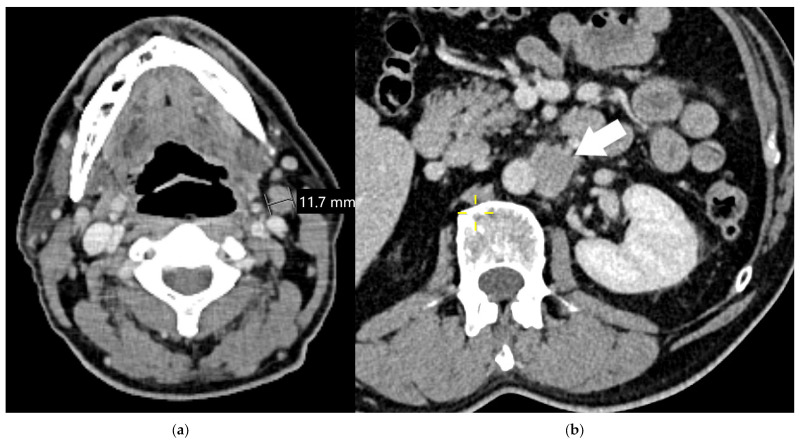
Extrathoracic lymph node metastases are considered to be distant metastases. Enlarged left-sided cervical level II lymph node (**a**); enlarged retroperitoneal lymph node (arrow, (**b**)).

At the time of initial diagnosis, about 40% of lung cancer patients have distant metastases, with the highest incidence observed in patients with SCLC [83]. The most common sites of extrathoracic lung cancer metastases are the brain, bones, liver, and adrenal glands. Furthermore, involvement of non-regional extrathoracic lymph nodes, such as distant neck or abdominal lymph nodes, falls under the same category, so both non-regional intrathoracic and extrathoracic lymph node involvement indicates metastatic disease (Figure 18). The M1b category represents a single metastasis involving an extrathoracic organ or non-regional lymph node. In the M1c1 category, multiple metastases involve a single extrathoracic organ system regardless of the total number of lesions, whereas in the M1c2 category, there are multiple extrathoracic metastases in multiple organ systems. It is important to note that the organ system may be a solitary organ, paired organ, or diffusely spread throughout the body; for example, the skeleton is considered to be one organ. In the M1c1 category, cancer has spread but is limited to one organ system, allowing for potential surgical intervention or ablation therapies in instances of oligometastatic disease. However, the M1c2 category refers to cases where metastases involve at least two or more organ systems. This more extensive spread typically leads to a worse prognosis and often necessitates systemic therapies, including chemotherapy or immunotherapy [78]. Imaging is crucial for assessing the extent of metastatic disease, leading to customized and effective treatment strategies [82].

The combination of CT, MRI, and FDG PET/CT is essential for evaluating metastatic spread in lung cancer. CT is excellent for visualizing anatomical structures and identifying large metastatic lesions, while MRI excels in assessing soft-tissue involvement, particularly in the brain and bones. PET/CT, on the other hand, offers functional imaging that highlights areas of increased metabolic activity, which is characteristic of cancer and is particularly valuable in detecting smaller or subclinical metastatic lesions that might be missed by anatomical imaging alone [84]. PET/CT may detect unsuspected metastases in up to 28% of patients with NSCLC, and it also has an impact on their management in up to 53% of patients [85]. By combining these modalities, clinicians can obtain a comprehensive view of the disease’s extent, guiding treatment decisions such as surgical intervention, radiation therapy, or systemic therapies like chemotherapy or immunotherapy.

**Figure 19:**
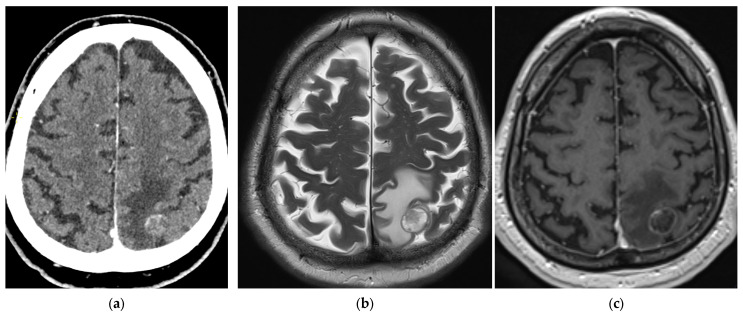
Brain metastasis: A single brain metastasis in the left parietal lobe in a patient with lung cancer indicates M1b disease. Axial post-contrast CT image (**a**), as well as T2-weighted (**b**) and post-contrast T1-weighted MRI images (**c**), showing a heterogeneous, rim-enhancing mass with a perifocal vasogenic edema.

The brain is one of the most common sites for the spread of lung cancer [86]. Studies have shown that 1.6–21% of asymptomatic stage III lung cancer patients harbor undiagnosed brain metastases [87,88,89]. SCLC patients have the highest risk of brain metastases [90]. MRI with a gadolinium-based contrast agent is the preferred imaging method for detecting the presence and the number of brain metastases, due to its superior soft-tissue contrast [91]. It has improved sensitivity as compared to CT and the ability to visualize small lesions in the brain parenchyma, especially if 3-Tesla scanners are used [92]. If no MRI scanners are available, or if there are contraindications for an MRI examination, a contrast-enhanced brain CT examination is an adequate substitute. Brain imaging should be performed in all lung cancer patients presenting with neurological symptoms or signs. According to the ESMO guidelines, a contrast-enhanced MRI or CT should also be carried out at diagnosis in all patients with metastatic NSCLC, and it may be considered in patients with early and locally advanced NSCLC considered for curative therapy [29,84]. Furthermore, brain imaging, preferably MRI, is recommended in all SCLC patients with localized disease and in stage IV SCLC patients not undergoing prophylactic cranial irradiation [93]. The ASCO also advises brain imaging for patients with clinical stage III NSCLC [25]. Lung cancer metastases in the brain are commonly multiple and may be associated with leptomeningeal disease, especially in patients with adenocarcinoma [92]. They have variable signal intensity on T2-weighted images and show spherical or ring contrast enhancement with vasogenic edema in the surrounding white matter (Figure 19). FDG PET/CT is insufficiently specific for the detection of brain metastases due to the large physiological FDG uptake of the brain [87].

**Figure 20:**
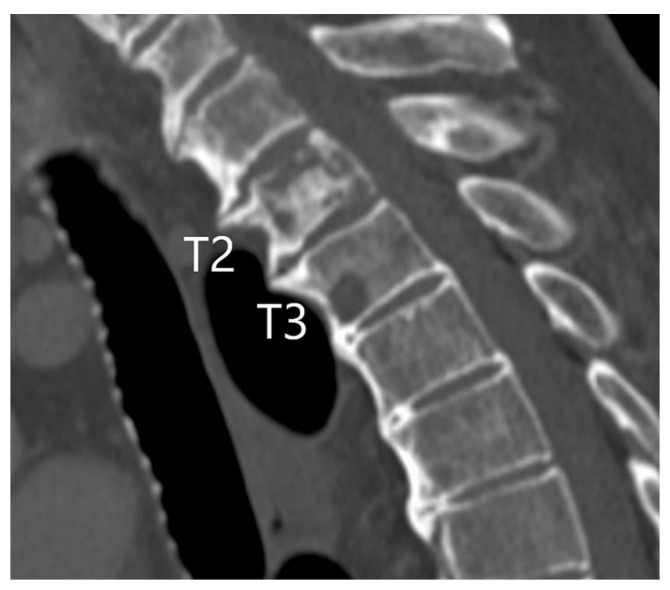
M1c1 category: A sagittal reformatted CT image (bone window) showing lytic metastasis in the T3 vertebral body, with mixed lytic and sclerotic metastatic lesions in the T2 vertebral body. No other distant metastases were evident.

**Figure 21:**
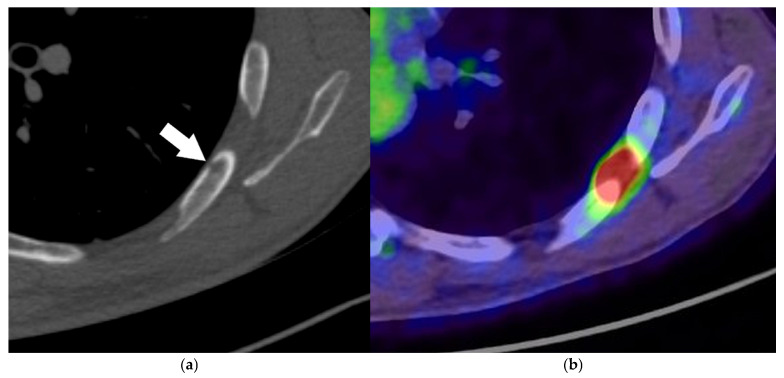
PET/CT bone metastasis detection: Bone window CT scan (width 1800, level 400) showing a subtle rib lytic lesion (arrow) that was not detected during CT analysis (**a**). High FDG uptake in the bone lesion is evident on PET/CT, confirming bone metastasis (**b**).

About 30–40% of patients with lung cancer develop bone metastases, and the most commonly affected bone is the spine, followed by the ribs and pelvic bones [94]. Lung cancer bone metastases have a variable lytic, sclerotic, or mixed appearance (Figure 20). They may be assessed using a combination of advanced imaging techniques to identify and evaluate the extent of the disease. However, the commonly used CT has limited accuracy for the detection of bone metastases, with a sensitivity of 74% and specificity of 56% [95]. False positive CT findings include bone islands and intraosseous hemangiomas, but they can usually be recognized by very high attenuation values and a polka dot sign, respectively [37]. FDG PET/CT is a highly sensitive method for detecting metabolically active bone metastases (Figure 21) [96]. MRI is another important tool offering detailed anatomical insights for evaluating bone marrow involvement and soft-tissue extension. It also allows the detection of smaller bone lesions and epidural invasion, which may cause neurologic deficit [97]. Bone scintigraphy with technetium-99m-labeled compounds remains a traditional and effective approach for detecting widespread skeletal metastases. Additionally, dual-energy computed tomography (DECT) improves lesion characterization by differentiating metastatic lesions from benign conditions such as fractures or degenerative changes based on material-specific attenuation [98]. All of these modalities are often complementary, improving diagnostic accuracy and guiding appropriate management. While monitoring patients after therapy for bone metastases, imaging modalities like CT or PET/CT can show the transformation of osteolytic metastatic lesions into osteosclerotic bone-forming lesions, representing a complex biological response, often indicative of effective treatment [99]. Therefore, the transformation of osteolytic metastatic lesions into osteosclerotic ones, as a good response to therapy, should not be misinterpreted as new osteoblastic metastases.

The liver and adrenal glands are also common sites for metastasis in lung cancer, especially in advanced NSCLC. CT and PET/CT are the preferred imaging techniques for assessing liver and adrenal metastasis. While CT provides detailed anatomical images to detect liver and adrenal masses, PET/CT provides functional imaging by identifying areas of increased glucose metabolism, typical of cancer cells.

**Figure 22:**
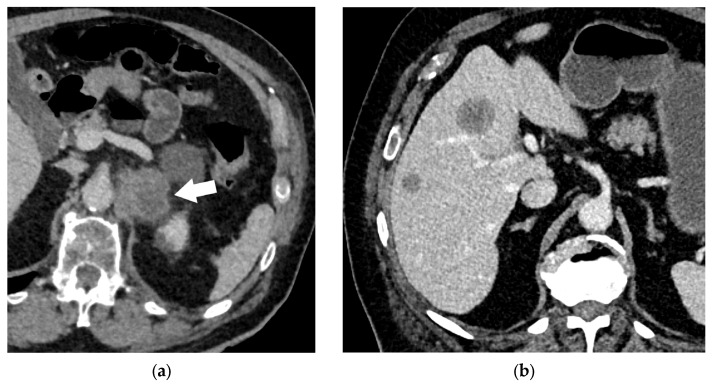
M1c2 category: Axial contrast-enhanced CT scan showing a strongly enhancing, heterogeneous, ill-defined mass in the left adrenal gland, indicative of a metastatic lesion (**a**). Multiple hypoattenuating liver lesions in portal venous phase CT suggest hepatic metastases (**b**).

**Figure 23:**
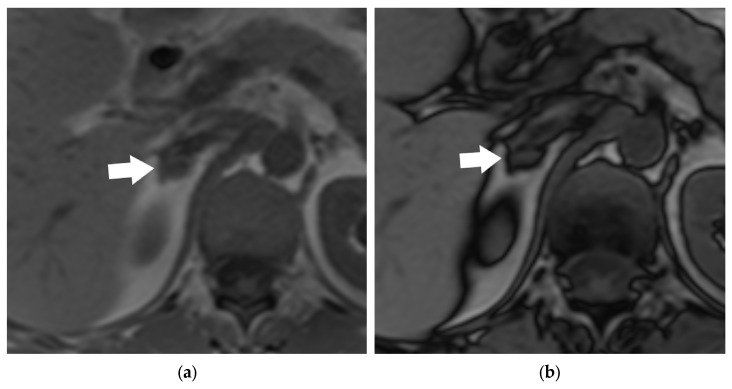
Chemical-shift MRI characterization of adrenal mass: Axial in-phase T1-weighted MRI image showing a mass (arrow) in the right adrenal gland (**a**). There is no signal loss of the mass (arrow) on the out-of-phase T1-weighted image (**b**), suggesting an adrenal metastasis over an adrenal adenoma in a patient with lung cancer.

Adrenal metastases should be differentiated from other adrenal masses, especially from adrenal adenoma, which is common in older individuals, occurring in up to 7% of individuals older than 70 years [100]. On CT, adrenal metastases appear as focal, heterogeneous, ill-defined masses with strong contrast enhancement on the portal venous phase and slower washout than adenomas (Figure 22a). PET/CT helps to differentiate between benign and malignant lesions based on metabolic activity, providing a more reliable assessment of metastatic involvement [27]. When using a threshold of ≤10 Hounsfield units to identify benign adrenal adenomas on the non-contrast CT component of FDG PET/CT, the sensitivity, specificity, and positive and negative predictive values reach 100%, 98%, 97%, and 100%, respectively [101]. Furthermore, the increasingly used DECT may differentiate lung cancer adrenal metastases from adrenal adenoma, even if post-contrast images were acquired only to reduce the radiation dose. Using DECT, it is possible to measure lesion attenuation values on virtual non-contrast images, enabling the detection of lipid-rich adenomas with low attenuation values (<10 Hounsfield units) and a higher fat fraction. Furthermore, metastases show higher iodine density than adrenal adenomas, improving diagnostic accuracy and reducing unnecessary procedures [102]. MRI may be used as a second-line method for the further characterization of adrenal lesions (Figure 23) [103].

Although an abdominal contrast-enhanced CT scan in the portal venous phase is a part of the standard imaging evaluation for lung cancer staging, there are often inconclusive findings of liver lesions that can mimic metastases (e.g., hemangioma), suggesting further workup (Figure 22b). Lung cancer metastases typically appear as hypovascular liver lesions, and rarely as hyperattenuating lesions, mainly in cases of neuroendocrine tumors [104,105]. In case of inconclusive single-phase CT findings, a multiphase contrast-enhanced liver CT, MRI, and/or PET/CT may help in further lesion characterization. Liver MRI is particularly imposed by its high sensitivity and specificity for distinguishing benign liver lesions from metastases and the possibility of detecting small metastases below the threshold of resolution of a CT examination [106,107].

## 6. Conclusions

- The detailed knowledge of the updated TNM system with its imaging correlation is essential for staging lung cancer, including NSCLC, SCLC, and lung neuroendocrine neoplasms. An accurate radiological report is critical for accurate clinical staging and guiding treatment strategies to optimize outcomes.
- Contrast-enhanced CT is a fundamental tool in the diagnosis and staging of lung cancer, but its sensitivity for detecting local invasion and regional or distant metastases can be suboptimal compared to advanced modalities.
- FDG PET/CT offers improved sensitivity and specificity over CT but is not effective for cancers with low FDG avidity or for the detection of brain metastases. PET/CT can also produce false positive results in conditions like infections or inflammatory diseases that are FDG-avid.
- Familiarity with the capabilities and limitations of each imaging modality is essential for accurate TNM staging. A multimodal imaging approach, combined with a deep understanding of the TNM classification system and potential diagnostic challenges, is key to ensuring precise lung cancer staging and effective patient management.

## Figures and Tables

**Table 1:** The advantages and potential limitations of PET/CT and MRI in lung cancer staging.

Imaging Method	Advantages	Limitations
PET/CT	High sensitivity for distant metastases High sensitivity for hilar and mediastinal lymph node metastases	Low sensitivity for detection of brain metastases due to a high physiological glucose uptake in the brain Limited effectiveness for low-FDG-avidity cancers (e.g., carcinoid tumors, indolent lung adenocarcinomas) FDG avidity of lesions unrelated to lung cancer (e.g., inflammation, infection, other benign and malignant tumors)
MRI	Superb soft-tissue contrast resolution enabling differentiation between the T3 and T4 categories in Pancoast tumors due to better evaluation of cervical and thoracic nerve roots, brachial plexus, or stellate ganglion invasion High sensitivity for detection of metastases in the central nervous system	Lower availability Contraindications for MRI examinations Long-lasting examinations Patient cooperation required

FDG—fluorodeoxyglucose, PET/CT—fluorodeoxyglucose positron emission tomography/computed tomography, MRI—magnetic resonance imaging.

**Table 3:** Recommendations for tumor size measurement on CT examination, applicable for lung cancer staging based on the TNM staging system, ninth edition.

Parameter	Recommendations
Slice thickness	Use contiguous thin sections (≤1 mm)
Measurement settings	Perform all measurements using a lung window setting (width 1500, level −600) with a sharp filter
Recording dimensions	Document nodule dimensions in centimeters, including millimeter increments
Solid and pure ground-glass nodules	Measure the maximum long-axis diameter in the axial, sagittal, and coronal planes
Part-solid nodules	Measure the maximum long-axis diameter of the nodule, including the ground-glass (GG) component, in the axial, sagittal, and coronal planes. Also, measure the maximum long-axis dimension of the largest solid component. Report both dimensions; however, for staging purposes, use only the largest solid component dimension

**Table 4:** The International Association for the Study of Lung Cancer (IASLC) lymph node map.

Station	Anatomical Region	Borders on a CT Scan [73]
1R and 1L	Lower cervical, supraclavicular, and sternal notch nodes	The lower margin of the cricoid cartilage (superiorly), clavicles and the upper border of the manubrium (inferiorly), the midline of the trachea serves as the border between 1R and 1L
2R and 2L	Upper paratracheal nodes	Bilateral superior border: apex of the right lung and pleural space, and in the midline, the upper border of the manubrium 2R inferior border: the intersection of the caudal margin of the left brachiocephalic (innominate) vein with the trachea 2L inferior border: superior border of the aortic arch The border between 2R and 2L is the left lateral border of the trachea
3	Pre-vascular nodes (3a) Retrotracheal nodes (3b)	Pre-vascular: chest apex (superiorly), level of carina (inferiorly), posterior aspect of sternum (anteriorly), anterior border of superior vena cava (posteriorly, right side), left carotid artery (posteriorly, left side) Retrotracheal: chest apex (superiorly), level of carina (inferiorly) Midline nodes are considered to be ipsilateral
4R and 4L	Lower paratracheal nodes	4R: the intersection of the caudal margin of the left brachiocephalic (innominate) vein with the trachea (superiorly), lower border of azygos vein (inferiorly) 4L: superior border of the aortic arch (superiorly), upper rim of the left main pulmonary artery (inferiorly), ligamentum arteriosum (laterally) The border between 4R and 4L is the left lateral border of the trachea
5	Subaortic nodes	Ligamentum arteriosum and aorta (medially), the lower border of the aortic arch (superiorly), the upper rim of the left main pulmonary artery (inferiorly), proximal to the first branch of the left pulmonary artery
6	Para-aortic nodes	Anterior and lateral to the ascending aorta, aortic arch, and the brachiocephalic trunk (innominate artery). Superior border: the upper border of the aortic arch Inferior border: the lower border of the aortic arch
7	Subcarinal nodes	Superior border: the carina Inferior border: the lower border of the bronchus intermedius (right side), the upper border of the lower lobe bronchus (left side)
8	Paraesophageal nodes	Adjacent to the esophageal wall to the right or left of the midline, excluding subcarinal nodes Superior border: the lower border of the bronchus intermedius (right side), the upper border of the lower lobe bronchus (left side) Inferior border: the diaphragm
9	Pulmonary ligament	Within the pulmonary ligament Superior border: the inferior pulmonary vein Inferior border: the diaphragm
10	Hilar	Nodes adjacent to the mainstem bronchus, proximal portions of the pulmonary veins, and the pulmonary artery Superior border: the lower rim of the azygos vein (right side), the upper rim of the pulmonary artery (left side) Inferior border: interlobar region
11	Interlobar	Between origins of lobar bronchi
12	Lobar	Adjacent to lobar bronchi
13	Segmental	Adjacent to segmental bronchi
14	Subsegmental	Adjacent to subsegmental bronchi

R—right, L—left.

**Table 5:** N descriptors for the TNM-9 clinical classification of lung cancer.

Descriptor	Description [30]	Comment
Nx	Regional lymph nodes cannot be assessed	
N0	No regional lymph node metastasis	
N1	Metastasis(es) in ipsilateral peribronchial or ipsilateral hilar or intrapulmonary lymph nodes	Ipsilateral stations 10–14 [73] Direct extension of primary tumor included [30]
N2	Metastasis(es) in the ipsilateral mediastinal or subcarinal nodal station(s)	The border between 2R and 2L, as well as between 4R and 4L is the left lateral tracheal border For other stations, midline nodes are considered to be ipsilateral [73]
N2a	Metastasis(es) in a single ipsilateral mediastinal or subcarinal nodal station	Includes metastasis(es) both in single or multiple lymph nodes in the same single nodal station
N2b	Metastases in multiple ipsilateral mediastinal nodal stations with or without involvement of the subcarinal nodal station	
N3	Metastasis(es) in contralateral mediastinal, contralateral hilar, ipsilateral or contralateral scalene or supraclavicular lymph node(s)	Metastases to non-regional intrathoracic lymph nodes are considered distant metastases [24,27,76]

**Table 6:** M descriptors for the TNM-9 clinical classification of lung cancer.

Descriptor	Description [30]	Comment
M0	No distant metastasis	
M1	Distant metastasis(es)	
M1a	Pleural or pericardial nodules or malignant pleural or pericardial effusions Separate tumor nodule(s) in a contralateral lobe	The pleural or pericardial effusions should be excluded as staging descriptors if multiple microscopic fluid examinations are negative for tumor and the fluid is non-bloody and is not an exudate [30]
M1b	Single extrathoracic metastasis in a single organ system	Includes involvement of a single non-regional intrathoracic or extrathoracic lymph node [24,27,30,76]
M1c1	Multiple extrathoracic metastases in a single organ system	The organ system may be a solitary organ, paired organ, or diffuse throughout the body. The skeleton is considered to be one organ [82]
M1c2	Multiple extrathoracic metastases in multiple organ systems	

## Data Availability

No new data were created or analyzed in this study. Data sharing does not apply to this article.
